# The Nim1 kinase Gin4 has distinct domains crucial for septin assembly, phospholipid binding and mitotic exit

**DOI:** 10.1242/jcs.183160

**Published:** 2016-07-15

**Authors:** Jie Ying Au Yong, Yan-Ming Wang, Yue Wang

**Affiliations:** 1 Institute of Molecular and Cell Biology, Agency for Science, Technology, and Research, Singapore 138673; 2 Department of Biochemistry, Yong Loo Lin School of Medicine, National University of Singapore, Singapore 117597

**Keywords:** *Candida albicans*, Septin assembly, Nim1 kinases, Gin4, Cdc14

## Abstract

In fungi, the Nim1 protein kinases, such as Gin4, are important regulators of multiple cell cycle events, including the G2–M transition, septin assembly, polarized growth and cytokinesis. Compelling evidence has linked some key functions of Gin4 with the large C-terminal non-kinase region which, however, is poorly defined. By systematically dissecting and functionally characterizing the non-kinase region of Gin4 in the human fungal pathogen *Candida albicans*, we report the identification of three new domains with distinct functions: a lipid-binding domain (LBD), a septin-binding domain (SBD) and a nucleolus-associating domain (NAD). The LBD and SBD are indispensable for the function of Gin4, and they alone could sufficiently restore septin ring assembly in *GIN4-*null mutants. The NAD localizes to the periphery of the nucleolus and physically associates with Cdc14, the ultimate effector of the mitotic exit network. Gin4 mutants that lack the NAD are defective in spindle orientation and exit mitosis prematurely. Furthermore, we show that Gin4 is a substrate of Cdc14. These findings provide novel insights into the roles and mechanisms of Nim1 kinases in the regulation of some crucial cell cycle events.

## Abstract (precis)

**Summary:** Systematic dissection of the Gin4 kinase in the human pathogenic fungus *Candida albicans* uncovers three new functional domains that interact with distinct cellular components.

## INTRODUCTION

Cell division requires temporal precision in the sequential coordination of events that are monitored and enforced through surveillance mechanisms called checkpoints (Hartwell and Weinert, 1989; Lew and Burke, 2003; Murray, 1992; Rudner and Murray, 1996; Zhou and Elledge, 2000). In eukaryotes, although cyclin-dependent kinases (CDKs) are the master cell cycle regulators (Morgan, 1997; Murray, 1994, 2004; Walworth, 2000), several other protein kinases also play crucial roles in governing various cell cycle events. One example is the Nim1 serine/threonine protein kinases. In *Schizosaccharomyces pombe*, the Nim1 kinases Cdr1 and Cdr2 are essential components of the cell size checkpoint, which promote the G2–M transition by downregulating the CDK inhibitor Wee1 (Kanoh and Russell, 1998; Moseley et al., 2009). In *Saccharomyces cerevisiae* (*Sc*), a homologous kinase Hsl1 plays a similar role in the morphogenesis checkpoint (Crutchley et al., 2009). Nim1 kinases have also been intimately linked to the regulation of septin organization and dynamics, cytokinesis and morphogenesis (Altman and Kellogg, 1997; Barral et al., 1999; Li et al., 2012; Mortensen et al., 2002; Wightman et al., 2004). Fungal Nim1 kinases are large proteins of >1000 amino acids with a conserved N-terminal kinase domain followed by an extended poorly characterized C-terminal region (Akada et al., 1997; Asano et al., 2006; Longtine et al., 1998; Mortensen et al., 2002; Okuzaki et al., 1997; Wightman et al., 2004).

The Nim1 kinases are well-established regulators of septins in both *S. cerevisiae* and the pathogenic fungus *Candida albicans* (*Ca*) (Li et al., 2012; Longtine et al., 1998; Sinha et al., 2007). The filament-forming septins are GTP-binding proteins conserved evolutionarily from yeast to humans (Beise and Trimble, 2011; Fung et al., 2014) and have roles in diverse processes that range from cytokinesis to cell morphogenesis (Longtine et al., 1996; Trimble, 1999). In yeasts, septins form scaffolds that anchor several cell cycle regulators (Bridges and Gladfelter, 2015; Crutchley et al., 2009). In humans, septins are associated with several diseases, such as cancer (Fung et al., 2014; Hall and Russell, 2004). Septins exist as complexes that can organize into many different structures, such as oligomeric rods, filaments, rings, hourglass-shaped collars and gauzes (Barral and Kinoshita, 2008; Weirich et al., 2008). *S. cerevisiae* contains five mitotic septins [Cdc3, Cdc10, Cdc11, Cdc12 and Shs1 (also known as Sep7)], and the septin cytoskeleton undergoes phase-dependent organizational and dynamic changes during each cell cycle (Dobbelaere et al., 2003; Ong et al., 2014). Septins first form a cap at the nascent bud tip, which later transforms into a ring and then an hourglass-shaped collar at the bud neck. The hourglass collar persists throughout mitosis and splits into two rings during cytokinesis before disassembly (Longtine and Bi, 2003). These morphological changes coincide with the dramatic remodeling of septin-filament orientation and crosslinking (Ong et al., 2014). Currently, the mechanisms governing septin assembly, disassembly and remodeling remain unclear. In *S. cerevisiae*, the CDK Cdc28 directly phosphorylates several septin isoforms, including Cdc3 and Cdc11 (Asano et al., 2006; Dobbelaere et al., 2003; Mortensen et al., 2002; Sinha et al., 2007; Tang and Reed, 2002). Cdc28 also acts indirectly through the Cdc42 GTPase, which recruits septins to the incipient bud site (Iwase et al., 2006). Other septin-associated protein kinases, such as Gin4 and the p21-activated kinase Cla4, are also important septin regulators that target Cdc3, Cdc10, and Shs1 (Altman and Kellogg, 1997; Asano et al., 2006; Bouquin et al., 2000; DeMay et al., 2009; Merlini et al., 2012). The septin ring-to-hourglass transition possibly involves the Elm1 and Gin4 kinases because cells lacking either kinase fail to assemble the hourglass-shaped collar (Altman and Kellogg, 1997; Asano et al., 2006; Bouquin et al., 2000; Lee et al., 2002). Splitting of the hourglass-shaped collar is proposed to involve the mitotic exit network (MEN) (Cid et al., 2001; Lippincott et al., 2001).

Recent studies in pathogenic fungi have shed new light on our understanding of Nim1 kinases and septin regulation (Bridges and Gladfelter, 2014; Gladfelter, 2010; Gonzalez-Novo et al., 2008; Li et al., 2012; Sinha et al., 2007). *C. albicans* can grow as three distinct morphological forms: yeast, pseudohyphae and hyphae (Sudbery, 2011), and possesses orthologues of all *S. cerevisiae* septins (Warenda and Konopka, 2002). Septin organization and dynamics in *C. albicans* yeast and pseudohyphae resemble those of *S. cerevisiae* (Sudbery, 2001, 2011). However, *C. albicans* hyphae assemble septin structures with localizations and dynamics distinct from those in yeast cells (Gonzalez-Novo et al., 2008; Sudbery, 2001). *C. albicans gin4Δ/Δ* (null) mutants exhibit severe defects that are characterized by extreme bud elongation, and a failure in septin ring formation and cytokinesis (Li et al., 2012; Wightman et al., 2004). *Ca*Gin4 phosphorylates the septins Cdc11 and Sep7, regulating the yeast–hyphal transition and Sep7 dynamics, respectively (Li et al., 2012; Sinha et al., 2007). However, we have observed previously that *C. albicans* cells expressing a mutant Gin4 that lacks the kinase domain is able to assemble the septin ring at the bud neck and displays milder defects than the *gin4Δ/Δ* mutant, indicating that some important functions of Gin4 are furnished by regions outside the kinase domain (Li et al., 2012). Similar observations have been reported in *S. cerevisiae* strains expressing kinase-dead Gin4 (Longtine et al., 1998). However, the Gin4 non-kinase region remains poorly characterized, except for a phospholipid-binding KA1 domain found at the C-terminus of *S. cerevisiae* Nim1 kinases (Moravcevic et al., 2010).

In this study, we have performed a systematic dissection and functional characterization of the non-kinase region of *Ca*Gin4 and have uncovered new roles for Gin4 in the control of septin organization, septin interaction with the plasma membrane and mitotic exit. We define three new functional domains: a lipid-binding domain (LBD), a septin-binding domain (SBD) and a nucleolus-associating domain (NAD). The LBD and SBD are indispensable for the function of Gin4, and a fragment containing only the LBD and SBD is sufficient to support septin ring formation at the bud neck. The NAD mediates localization to the periphery of the nucleolus and physically associates with Cdc14. Deleting the NAD causes defects in spindle orientation and premature mitotic exit. We also show that Gin4 is a substrate of Cdc14.

## RESULTS

### Systematic dissection of the non-kinase domain of *Ca*Gin4

**Fig. 1.**
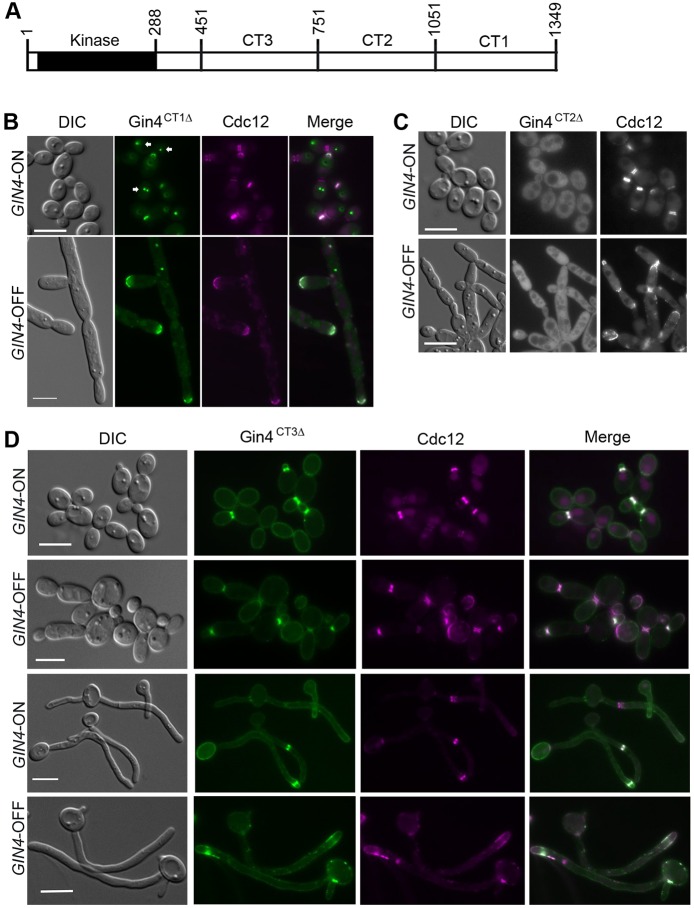
**CT1 and CT2 are indispensable for Gin4 function.** (A) Schematic representation of *Ca*Gin4 domain organization and construction of truncation mutants. Numbers indicate amino acid residues. (B) Phenotype of *gin4^CT1Δ^*-expressing cells (called JY8) under *GIN4-*ON and -OFF conditions. *GIN4*-ON cells were grown in MalMM and *GIN4*-OFF cells in GMM, respectively, at 30°C to log phase. Cells were examined by using differential interference contrast (DIC) and fluorescence microscopy. Arrows point to dots formed by GFP–Gin4^CT1Δ^. (C) Phenotype of *gin4^CT2Δ^*-expressing cells (called JY31). Cells were grown and examined as described in B. (D) Phenotype of *gin4^CT3Δ^*-expressing cells (called JY49). For yeast cells, cells were grown as described in A. For hyphal growth, the yeast cells were induced for hyphal growth with 20% serum (FCS) at 37°C for 2 h. Scale bars: 5 µm.

*Ca*Gin4 comprises 1349 amino acids with the kinase domain (residues 28–288) located near to the N-terminus. To identify functional domains in the non-kinase region (residues 289–1349), we first dissected it into three 300-amino-acid segments, CT1 (residues 1051–1349), CT2 (residues 751–1050) and CT3 (residues 451–750) (Fig. 1A). Two sets of truncated alleles were constructed – one set in which each of the CT segments had been deleted (*gin4^CT1^*^Δ^, *gin4^CT2^*^Δ^ and *gin4^CT3^*^Δ^) and another in which each construct contained only CT1, CT2 or CT3. All the truncated alleles were expressed from the *MET3* promoter in a strain that carried a single copy of *GIN4* regulated by the *MAL2* promoter (*gin4*Δ/P*_MAL2_*-*GIN4*). The *MAL2* promoter allows *GIN4* expression (*GIN4*-ON) in medium with maltose as the sole carbon source and *GIN4* repression (*GIN4*-OFF) in medium containing glucose. The *GIN4*-ON cells were indistinguishable from the wild-type (WT) cells, whereas the *GIN4*-OFF cells phenocopied the *gin4Δ/Δ* mutant. Expressing WT *GIN4* from the *MET3* promoter fully rescued the defects of the *GIN4*-OFF cells, whereas switching off the *MET3* promoter led to a phenotype matching that of *gin4Δ/Δ* mutants. Thus, the *gin4*Δ/P*_MAL2_-GIN4* strain allowed us to investigate each *gin4* allele in both *GIN4*-ON and -OFF backgrounds. All the Gin4 proteins carried an N-terminal GFP tag, unless indicated otherwise.

We first examined the cellular localization of Gin4^CT1Δ^, Gin4^CT2Δ^ and Gin4^CT3Δ^ by performing fluorescence microscopy. *GIN4*-ON yeast cells that expressed Gin4^CT1Δ^ showed no discernible defects, and GFP–Gin4^CT1Δ^ colocalized with Cdc12–mCherry at the bud neck throughout the cell cycle (Fig. 1B, top). However, under *GIN4*-OFF conditions, the *gin4^CT1^*^Δ^ cells exhibited morphological defects similar to the *gin4Δ*/*Δ* cells in which no septin ring was formed, and GFP–Gin4^CT1Δ^ colocalized with Cdc12–mCherry to pseudohyphal tips (Fig. 1B, bottom), indicating that *gin4^CT1^*^Δ^ cannot support septin ring formation and that CT1 is essential for Gin4 function. The ability of Gin4^CT1Δ^ to colocalize with the septins both at the bud neck in *GIN4*-ON cells and at the pseudohyphal tips in *GIN4*-OFF cells suggests that CT1 is not required for Gin4 to associate with septin complexes. Surprisingly, in *GIN4*-ON cells, GFP–Gin4^CT1Δ^ often appeared as a single dot in both the mother and daughter compartments or as a pair of closely positioned dots in the mother compartment (Fig. 1B), reminiscent of spindle pole bodies (SPBs).

*GIN4*-ON yeast cells that expressed Gin4^CT2Δ^ showed normal morphology and normal septin rings marked by Cdc12–mCherry (Fig. 1C). However, GFP–Gin4^CT2Δ^ did not localize to the bud neck; instead, it was detected in the entire cytoplasm. Also, when *GIN4* expression was repressed, *gin4^CT2^*^Δ^ cells developed a pseudohyphal morphology that was, however, considerably less severe than that of *gin4Δ/Δ* cells. The pseudohyphae were shorter and had sharper septal constrictions, in which GFP–Gin4^CT2Δ^ showed the same cytoplasmic localization. Septins, mostly in the form of abnormal rings or aggregates, appeared in the septal region in ∼47% of the cells and as a broad crescent at pseudohyphal tips. The data suggest that CT2 might contain motifs required for Gin4 to associate with and facilitate the assembly of septin complexes.

*GIN4*-ON yeast cells expressing Gin4^CT3Δ^ showed normal morphology and septin rings (Fig. 1D, top), and GFP–Gin4^CT3Δ^ colocalized properly with septins to the bud neck. GFP–Gin4^CT3Δ^ also showed weak localization throughout the entire plasma membrane. Under *GIN4*-OFF conditions, the majority of the cells were of the yeast morphology but were obviously larger than *GIN4*-ON cells, and ∼30% of these cells were moderately elongated (Fig. 1D, top). In these cells, septin rings were assembled and localized normally at the bud neck throughout the cell cycle, and cytokinesis occurred successfully. As *gin4Δ/Δ* pseudohyphae do not respond to hyphal induction (Wightman et al., 2004), we tested whether *gin4^CT3^*^Δ^ yeast cells could develop hyphae upon serum induction. We found that *GIN4*-OFF *gin4^CT3^*^Δ^ cells grew true hyphae (Fig. 1D, bottom). However, Gin4^CT3Δ^ often formed diffuse bands behind the hyphal tip or random cortical aggregates. Also, septin rings failed to form at the septum; instead, they mislocalized with Gin4^CT3Δ^, suggesting that CT3 might be required for hyphal-specific septin ring assembly.

### CT1 contains an LBD, which mediates the association with the plasma membrane

**Fig. 2.**
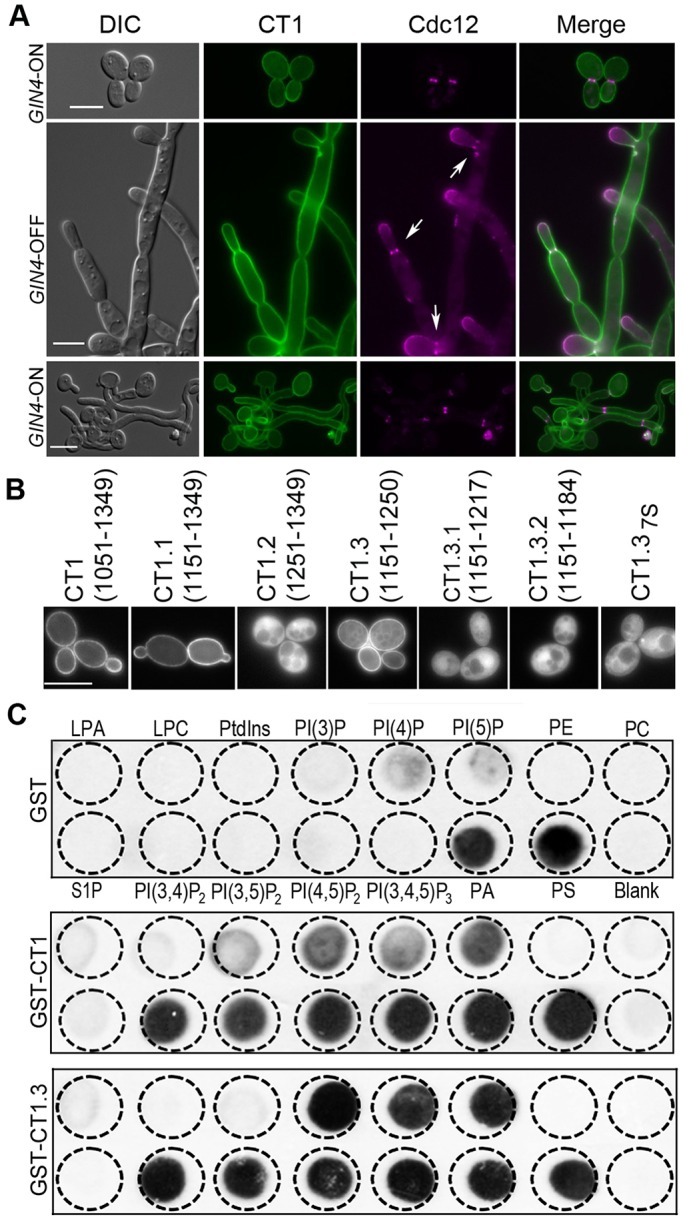
**CT1 contains the domains required for phospholipid binding and localization to the plasma membrane.** (A) Phenotype of CT1-expressing cells (called JY9). Yeast and hyphal cells were grown and examined as described in Fig. 1. Arrows point to septin rings at the junction between a new bud and the mother cell. (B) CT1.3 (residues 1151–1250) mediates the localization at the plasma membrane. CT1 was truncated into smaller fragments, each tagged with GFP at the N-terminus and expressed in strain LCR43. All seven basic residues were replaced with serine in CT1.3 to yield CT1.3_7S_ (called JY23). (C) Purified GST–CT1 and GST–CT1.3 bind to phosphoinositides *in vitro*. GST, GST–CT1 and GST–CT1.3 expressed and purified from *E. coli* were tested for their ability to bind phospholipids by using the PIPstrips™. LPA, lysophosphatidic acid; LPC, lysophosphocholine; PA, phosphatidic acid; PC, phosphatidylcholine; PE, phosphatidylethanomaline; PS, phosphatidylserine; S1P, sphingosine 1-phosphate. Scale bars: 5 µm.

We next examined the cellular localization of CT1. *GIN4*-ON cells expressing CT1 were normal in both yeast and hyphal growth; and strikingly, GFP–CT1 localized to the entire plasma membrane (Fig. 2A). In comparison, *GIN4*-OFF CT1 cells grew as pseudohyphae with cytokinetic defects, and GFP–CT1 also showed the same plasma membrane localization. Interestingly, we observed that a faint septin ring often existed transiently at the base of newly emerged buds, suggesting CT1 could partially facilitate septin ring assembly.

While our work was in progress, Moravcevic et al. (2010) identified a ∼100-amino-acid kinase-associated-1 (KA1) domain at the C-terminus of three *S. cerevisiae* Nim1 kinases – Gin4, Kcc4, and Hsl1 – and found that the KA1 domain mediates plasma membrane association through phospholipid binding. *C. albicans* has orthologues of *Sc*Gin4 and *Sc*Hsl1. Except for the kinase domain, *Ca*Gin4 and *Ca*Hsl1 share poor sequence similarity with their *S. cerevisiae* counterparts. Alignments of *Ca*Gin4 CT1 with the KA1 domains of *Sc*Gin4, *Sc*Kcc4, and *Sc*Hsl1 revealed substantial homology in the last 99 residues of *Ca*Gin4 (residues 1251–1349), suggesting that *Ca*Gin4 also contains a KA1 domain. However, the pairs of basic residues in the *Sc*Kcc4 KA1 domain required for plasma membrane association are not conserved in *Ca*Gin4. To determine whether the *Ca*Gin4 KA1 domain associates with the plasma membrane, we truncated CT1 to CT1.1 (residues 1151–1349) and CT1.2 (KA1; residues 1251–1349), and GFP-tagged each for expression in *gin4*Δ*/*P*_MAL2_*-*GIN4* cells. GFP–CT1.1 was found to localize to the plasma membrane, whereas the KA1 fragment localized in the cytoplasm. Therefore, the plasma-membrane-targeting residues lie not in KA1 but in residues 1151–1250. Indeed, the 1151–1250 fragment (CT1.3) localized to the plasma membrane. The plasma membrane localization was abolished with further truncation of CT1.3 (CT1.3.1 and CT1.3.2) (Fig. 2B). Next, we determined if the basic residues (K1163, K1166, K1167, R1190, K1191, K1197 and K1198) within CT1.3 are required for its plasma membrane localization. Unlike *Sc*Kcc4 KA1 and human MARK1 KA1 domains, in which mutating one of several pairs of basic residues is sufficient to abolish the membrane association (Moravcevic et al., 2010), plasma membrane localization was completely abolished only when all seven residues in CT1.3 were replaced with serine (CT1.3_7S_) (Fig. 2B). Also, unlike *GIN4*-OFF CT1 cells (Fig. 2A), *GIN4*-OFF CT1.3_7S_ cells did not support the formation of transient septin rings (Fig. S1A).

We next investigated whether CT1 and CT1.3 can associate with phospholipids. We purified GST–CT1 and GST–CT1.3 from *Escherichia coli* and tested their ability to bind to an array of phospholipids using PIPstrips™ (Fig. 2C). Purified GST was included as the negative control. CT1 exhibited specific affinity to phosphatidylinositol (PtdIns) and phosphoinositides, including phosphatidylinositol 3-phosphate [PI(3)*P*], phosphatidylinositol 4-phosphate [PI(4)*P*], phosphatidylinositol 5-phosphate [PI(5)*P*], phosphatidylinositol 3,4-bisphosphate [PI(3,4)*P*_2_], phosphatidylinositol 3,5-bisphosphate [PI(3,5)*P*_2_], phosphatidylinositol 4,5-bisphosphate [PI(4,5)*P*_2_] and phosphatidylinositol 3,4,5-trisphosphate [PI(3,4,5)*P*_3_]; and the affinity of CT1 for phosphoinositides seemed to increase with the number of negatively charged phosphates. CT1.3 exhibited affinity for the same set of phospholipids as CT1 except for PtdIns, to which CT1.3 does not bind (Fig. 2C). Our data demonstrate that the *Ca*Gin4 CT1.3 region possesses the phospholipid-binding ability and mediates the association of CT1 with the plasma membrane.

### CT2 contains an SBD

**Fig. 3.**
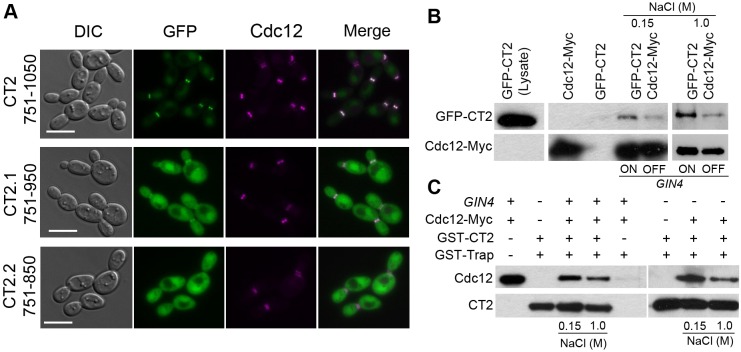
**CT2 localizes to the bud neck and interacts with septins.** (A) CT2 and two sub-fragments (2.1 and 2.2) tagged with N-terminal GFP were expressed in *gin4*Δ/P*_MAL2_-GIN4* cells that coexpressed Cdc12–mCherry (called JY35, JY37 and JY39, respectively). Cells were grown in *GIN4*-ON conditions at 30°C for microscopy analysis. (B) Coimmunoprecipitation of CT2 with Cdc12. C-terminally Myc-tagged Cdc12 was expressed in *gin4*Δ/P*_MAL2_-GIN4* cells that coexpressed GFP–CT2 (called JY40); *gin4*Δ/P*_MAL2_-GIN4* cells that expressed Cdc12–Myc (called JY69) alone were included as a negative control. Middle panel, cells were grown under *GIN4-*ON or -OFF conditions at 30°C to log phase. Coimmunoprecipitation was performed with anti-Myc beads and washed with buffer containing 150 mM NaCl, followed by western blot analysis to detect GFP or Myc. Right panel, immunoprecipitated anti-Myc beads were washed with buffer containing 1 M NaCl before western blot analysis. (C) Purified GST–CT2 pulls down Cdc12 from *C. albicans* cell lysates. GST–CT2 that had been purified from *E. coli* was incubated with GST-Trap beads. GST-Trap beads with or without bound GST–CT2 were mixed and incubated with *C. albicans* cell lysates expressing Cdc12–Myc (JY69). After washes with buffer containing 150 mM or 1 M NaCl, proteins on the beads were probed for Myc or GST by western blotting. Scale bars: 5 µm.

The inability of Gin4^CT2Δ^ to colocalize with the septins (Fig. 1C) suggests that CT2 might mediate the interaction with septins. Consistent with this hypothesis, CT2 colocalized with the septins at the bud neck in *GIN4*-ON cells throughout the cell cycle (Fig. 3A), and CT2 was lost from the bud neck when the septin ring split. Truncating CT2 by 100 amino acids from the C-terminus (CT2.1) substantially weakened its localization at the bud neck and caused significant cytoplasmic localization, and further truncation of another 100 amino acids (CT2.2) completely abolished the bud neck localization, suggesting that the entire 300 amino acids of CT2 are required for localization at the bud neck. To detect the interaction of CT2 with septins, coimmunoprecipitation was performed in *GIN4*-ON and -OFF cells by coexpressing Cdc12–Myc with GFP–CT2. Cells expressing Cdc12–Myc or GFP–CT2 alone were used as positive and negative controls, respectively. Cdc12–Myc was precipitated using anti-Myc beads, and the protein-bound beads were washed with buffers containing either 150 mM or 1 M NaCl. Cdc12–Myc and GFP–CT2 were then probed with antibodies against Myc and GFP, respectively, in western blot analysis. GFP–CT2 was detected in cell lysates that expressed Cdc12–Myc and GFP–CT2 but not in those expressing Cdc12–Myc or GFP–CT2 alone (Fig. 3B). The CT2–Cdc12 interaction was detected in both *GIN4*-ON and -OFF cells, although more Cdc12 was detected in the former, possibly owing to the lack of septin ring assembly in the latter. The Gin4–septin interaction remained stable in 1 M NaCl (Li et al., 2012). Similarly, the CT2–Cdc12 interaction was detectable after washes with 1 M NaCl (Fig. 3B). To confirm that the Gin4–septin interaction is directly mediated by CT2, purified GST–CT2 from *E. coli* was used to pull down CT2-binding proteins in cell lysates from either *GIN4*-ON or -OFF cells that expressed Cdc12–Myc. Subsequent western blotting for Myc clearly detected Cdc12–Myc in the pulldown products of GST–CT2 from both *GIN4*-ON and -OFF cells, but not from the products pulled down with the anti-GST-beads alone (Fig. 3C). Again, the CT2–Cdc12 interaction remained intact after washes with 1 M NaCl. The results show that CT2 contains a septin-binding domain (SBD) that mediates the association of Gin4 with Cdc12.

### The CT2+1 fragment is sufficient to organize the septin ring at the bud neck

**Fig. 4.**
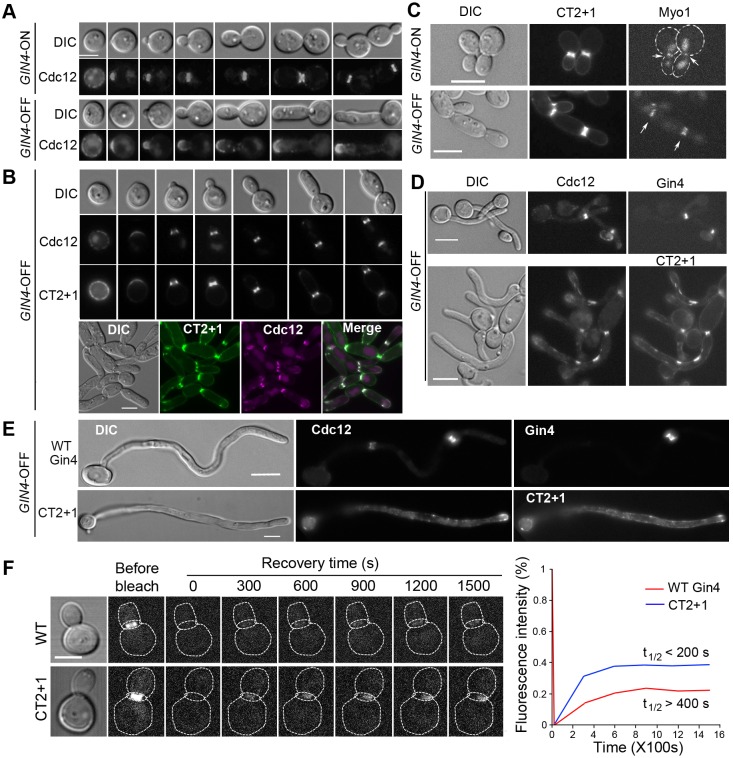
**The CT2+1 fragment can restore septin ring formation and localization to the bud neck in *GIN4*-OFF yeast cells.** (A) Cells expressing Cdc12–mCherry (called JY70) were cultured overnight under *GIN4*-ON conditions at 30°C. Elutriated G1 cells were released into *GIN4*-ON or *GIN4*-OFF conditions. Cells at different stages of budding were harvested for microscopy analysis. (B) G1 cells that coexpressed Cdc12–mCherry and GFP–CT2+1 (called JY71) were prepared as described above and released into *GIN4*-OFF conditions for further growth. Cells at different stages of bud growth (upper panel) and of an overnight culture (lower panel) were harvested for microscopy. (C) Cells that coexpressed Myo1–mCherry and GFP–CT2+1 (called JY73) were grown under *GIN4*-ON or *GIN4*-OFF conditions at 30°C to log phase before microscopy analysis. Arrows point to Myo1 at the bud neck. (D) Cells that coexpressed Cdc12–mCherry with GFP–Gin4 (called JY30) or GFP–CT2+1 (called JY71) were grown overnight under *GIN4-*OFF conditions before shifting to GMM+20% FCS for hyphal induction at 37°C for 2 h. (E) The same cells that are described in D after 3.5 h of hyphal induction. (F) FRAP analysis of the septin ring in cells that expressed WT Gin4 (JY70, *n*=13) or CT2+1 as the sole Gin4 source (JY71, *n*=12). Cells with a medium-sized bud were selected for FRAP, and the entire septin ring was bleached. Fluorescence recovery was measured at 5-min intervals after photobleaching. Average values were used to generate the curves. Scale bars: 5 µm.

We have reported previously that the Gin4 kinase domain is not required for septin ring assembly at the bud neck in *C. albicans* (Li et al., 2012). Here, we have identified an SBD that alone, however, cannot promote septin ring assembly. Also, we observed that expressing CT1 in *GIN4*-OFF cells supports the formation of transient septin rings, suggesting that CT1 might play a role in septin ring formation. Consistently, CT1 was found to coimmunoprecipitate with Cdc12 (Fig. S1B). Next, we asked if a fragment containing only the SBD and LBD is sufficient for septin ring assembly. To this end, GFP–CT2+1 (residues 751–1349) was coexpressed with Cdc12–mCherry in *GIN4*-ON and -OFF cells. To monitor septin assembly throughout a cell cycle, we collected *GIN4*-ON G1 cells, released them into *GIN4*-ON or -OFF conditions for further growth and harvested cells at intervals for examination under a microscope. *gin4*Δ*/P_MAL2_-GIN4* cells that expressed Cdc12–mCherry were used as a control. Upon release into the *GIN4*-ON medium, the control cells exhibited normal septin ring assembly, splitting and disassembly at the expected stages of the cell cycle (Fig. 4A, *GIN4*-ON). However, under *GIN4*-OFF conditions, Cdc12 formed a cap at the incipient bud tip, which persisted as the bud elongated, and the septin ring was never formed (Fig. 4A, *GIN4*-OFF). In contrast, in *GIN4*-OFF cells that expressed CT2+1, Cdc12 formed a ring at the bud neck and colocalized with CT2+1 throughout bud growth (Fig. 4B); however, the septin ring failed to split, and both CT2+1 and Cdc12 did not disassemble after cytokinesis. The *GIN4*-OFF CT2+1 cells grew into chains of moderately elongated yeast cells with septin rings persisting at the septum (Fig. 4B).

An important function of the septin ring is to recruit proteins such as Myo1, a component of the actomyosin ring, to the bud neck. Some septin mutations completely abolished the localization of Myo1 and actomyosin ring assembly at the bud neck (Finnigan et al., 2015; Lippincott and Li, 1998). To determine the functionality of the septin ring assembled in CT2+1 cells, we examined Myo1–mCherry localization in *GIN4*-ON and -OFF cells expressing CT2+1. Like in *GIN4*-ON cells, Myo1 and CT2+1 colocalized to the bud neck in *GIN4*-OFF cells, indicating that the assembled septin ring can recruit Myo1. However, Myo1 constriction and disassembly failed to occur in *GIN4*-OFF CT2+1 cells (Fig. 4C). Because *gin4Δ/Δ* pseudohyphae do not respond to hyphal induction (Wightman et al., 2004), we asked whether CT2+1 can restore hyphal growth in *gin4Δ/Δ* cells. In spite of the cytokinesis defects, expressing CT2+1 in *GIN4*-OFF cells produced separate yeast cells (Fig. S2). These cells responded to hyphal induction rather normally, producing long hyphae (Fig. 4D,E). Hyphal growth in the CT2+1 cells was not due to leaky expression of *GIN4* from the *MAL2* promoter because *GIN4*-OFF CT2+1-OFF cells were unresponsive to hyphal induction (Fig. S2). The results demonstrate that the CT2+1 region provides functions required for hyphal development. Interestingly, although CT2+1 and Cdc12 were often seen to colocalize at hyphal tips, septin rings were rarely found, even in long hyphae (Fig. 4E). In contrast to the ability of CT2+1 yeast cells to form septin rings, the lack of septin rings in CT2+1 hyphae indicates differential regulation of septin formation between yeast and hyphal growth. In yeast cells, septins within the ring undergo cell-cycle-dependent dynamic changes, which involve regulation by Gin4 (Dobbelaere et al., 2003). To examine whether the septin ring formation promoted by CT2+1 has altered dynamics, we performed fluorescence recovery after photo-bleaching (FRAP) analyses on Cdc12–mCherry in the septin ring. We examined cells with a medium-sized bud, in which the septin ring is immobile in WT cells (Dobbelaere et al., 2003). The entire septin ring was photobleached, and the fluorescence recovery monitored over time (Fig. 4F, left). In *GIN4*-ON WT cells, the fluorescence intensity recovered to ∼20% of the pre-bleached level, with a slow recovery rate of t_½_>400 s (*n*=13). In comparison, fluorescence recovery occurred twice as fast in *GIN4*-OFF CT2+1 cells (t_½_<200 s, *n*=12) and reached a higher level of ∼40%. Thus, septins in the ring that had assembled with the help of CT2+1 were more mobile than those in a normal ring. Taken together, the data demonstrate that the SBD-LBD region alone is sufficient to support the assembly of a partially functional septin ring.

### Identification of a NAD

**Fig. 5.**
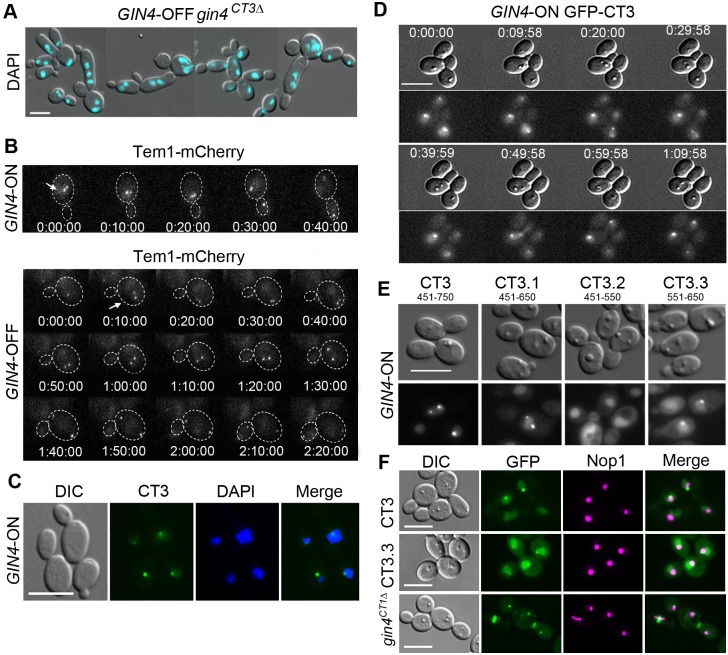
**CT3 contains a domain for localization to the nucleolus.** (A) *GIN4-*OFF *gin4^CT3*Δ*^* cells exhibited a multi-nucleated phenotype. Yeast cells of strain JY49 that had been grown under *GIN4*-OFF conditions were DAPI-stained to visualize the nuclei. (B) Time-lapse microscopy examination of the spindle in *GIN4*-OFF *gin4^CT3^*^Δ^ cells. Tem1 was C-terminally tagged with mCherry in *gin4*Δ/P*_MAL2_-GIN4* (called JY51) and *gin4*Δ/P*_MAL2_-GIN4 gin4^CT3^*^Δ^ (called JY50) cells. A representative *GIN4-*ON (upper panel) cell and a *GIN4*-OFF *gin4^CT3^*^Δ^ cell (JY50) are shown (time, h:min:sec). (C) CT3 localizes to the nucleus. *GIN4*-ON cells expressing GFP–CT3 (called JY43) were stained with DAPI before microscopy analysis. (D) Time-lapse microscopy examination of GFP–CT3 localization during a cell cycle in *GIN4*-ON GFP–CT3 cells (JY43). (E) Truncation of CT3 in order to locate the domain responsible for nucleolus foci localization. CT3 was truncated into CT3.1 (residues 451–650), CT3.2 (residues 451–550) and CT3.3 (residues 551–650). Each fragment was tagged with GFP at the N-terminus and expressed in *gin4*Δ/P*_MAL2_-GIN4* cells (called JY53, JY54 and JY55, respectively). Cells were grown in *GIN4*-ON conditions and examined with fluorescence microscopy. (F) CT3.3 colocalizes with Nop1. Nop1 was tagged with mCherry in cells that coexpressed GFP–CT3, GFP–CT3.3 and GFP–Gin4^CTΔ^ (called JY58, JY59 and JY60, respectively). Scale bars: 5 µm.

The larger sizes and mild elongation of *gin4^CT3Δ^* cells suggested possible mitotic defects. Indeed, many *gin4^CT3^*^Δ^ mother cells contained two or more well-separated nuclear masses (Fig. 5A), indicating nuclear division within the mother cell instead of across the bud neck as in WT cells. This phenotype is reminiscent of mutants defective in the spindle position checkpoint (SPC) in *S. cerevisiae* (Lew and Burke, 2003), suggesting that CT3 might contain signals necessary for cell cycle control. To further investigate the mitotic defects in *gin4^CT3Δ^* cells, we tagged Tem1, an SPB-associated GTPase, with mCherry as a cell cycle marker. Using time-lapse microscopy, we monitored Tem1–mCherry through one cell cycle in both *GIN4*-ON and *GIN4-*OFF *gin4^CT3^*^Δ^ cells. We recorded the time elapsed between the time when Tem1 first appeared as a pair of closely positioned dots (completion of SPB duplication) and that when one dot had just crossed the neck. Fig. 5B shows a typical *GIN4*-ON cell in which one Tem1 dot had already moved into the daughter cell 30 min after SPB duplication. In comparison, the two Tem1 dots in the *GIN4*-OFF *gin4^CT3Δ^* cell failed to align along the mother-bud axis and started to separate within the mother cell at 80 min (Fig. 5B). The spindle was fully elongated at 1 h 40 min within the mother cell, and the daughter-bound Tem1 dot finally crossed the neck at 2 h. In *GIN4*-ON cells, it took ∼24 min±10 min (*n*=10) for the daughter-bound Tem1 to enter the bud after SPB duplication, in contrast to ∼103 min±35 min (*n*=18) required in *gin4^CT3Δ^* cells. Despite normal septin ring localization and behavior, the *gin4^CT3Δ^* cells were defective in orientating the spindle poles along the mother-bud axis, leading to spindle elongation within the mother compartment. Our data suggest that CT3 might play a crucial role in positioning the spindle and activating mitotic exit.

We examined the subcellular localization of CT3 and found that GFP–CT3 appeared as one or two distinct dots in the nucleus in *GIN4*-ON cells (Fig. 5C) like GFP–Gin4^CT1Δ^ (Fig. 1B). Time-lapse monitoring through one cell cycle revealed that CT3 first appeared as a single dot, which later divided into two, with one migrating across the bud neck into the daughter cell (Fig. 5D). Moreover, GFP–CT3 never localized to the bud neck. To define the smallest domain responsible for this localization, we truncated CT3 to CT3.1 (residues 451–650), CT3.2 (residues 451–550) and CT3.3 (residues 551–650), and GFP-tagged each in *GIN4-*ON cells. GFP–CT3.3 behaved like CT3, whereas CT3.2 localized diffusely in the nucleus (Fig. 5E; Fig. S3A), indicating that CT3.3 contains the signal for the nuclear focal localization. Although the localization pattern is reminiscent of that of SPBs, GFP–CT3 and Tem1–mCherry signals did not overlap (Fig. S3B). Labeling the nucleolar protein Nop1 with mCherry revealed an association of CT3.3, CT3 and Gin4^CT1Δ^ dots with the periphery of the Nop1-labeled area (Fig. 5F). Therefore, we termed CT3.3 the nucleolus-associating domain (NAD).

### Cdc14 associates with the NAD and dephosphorylates Gin4

**Fig. 6.**
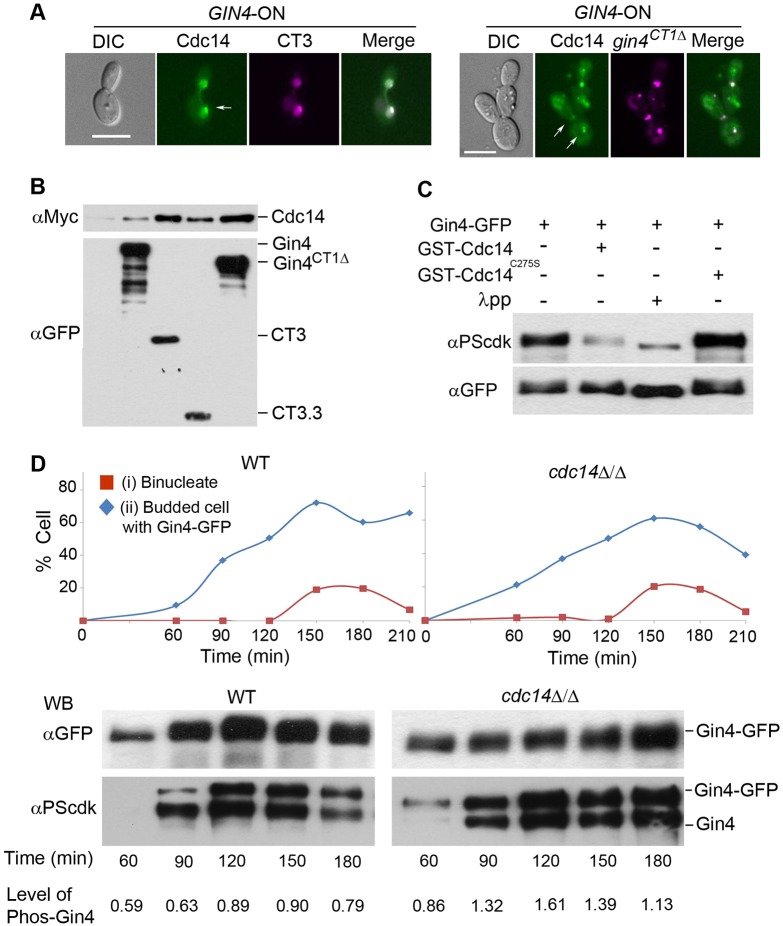
**Cdc14 physically associates with the NAD and dephosphorylates Gin4.** (A) Colocalization of CT3 and Cdc14. Cdc14–GFP was expressed from its endogenous promoter in *gin4*Δ/P*_MAL2_-GIN4* cells that coexpressed mCherry–CT3 (called JY62) or mCherry–Gin4^CT1Δ^ (called JY63). Cells were grown in *GIN4*-ON conditions and examined by performing fluorescence microscopy. (B) The NAD coimmunoprecipitates with Cdc14. Cdc14 was Myc-tagged C-terminally in *gin4*Δ/*P_MAL2_-GIN4* cells that coexpressed GFP–Gin4, GFP–CT3, GFP–CT3.3 and GFP–Gin4^CTΔ^ (called JY64, JY65, JY66 and JY67, respectively). Coimmunoprecipitation was performed in *GIN4*-ON cells by pulling down proteins using anti-GFP beads, followed by western blot analysis of the precipitates for Myc or GFP. (C) Cdc14 can dephosphorylate Gin4 *in vitro*. GST–Cdc14 and GST–Cdc14^C275S^ were purified from *E. coli* and mixed with immunopurified GFP–Gin4 (JY56) for *in vitro* phosphatase assays. Gin4 and phosphorylated Gin4 in the reaction products were detected by western blotting for GFP and PScdk, respectively. A λ-phosphatase-treated (λpp) sample was included as a positive control. (D) Involvement of Cdc14 in Gin4 dephosphorylation *in vivo*. Gin4–GFP was expressed from its endogenous promoter in WT (BWP17) and *cdc14Δ/Δ* cells that coexpressed Nop1–mCherry (called JY72 and JY68, respectively). Stationary-phase cultures were released into fresh medium for synchronous growth. Aliquots were collected at timed intervals for microscopy evaluation of cell cycle progression (*n*>100, upper panel). The rest of the cells were subjected to immunoprecipitation of GFP and western blot analysis for PScdk or GFP to reveal phosphorylated Gin4 and total Gin4–GFP (lower panel). Phosphorylated-Gin4–GFP signal (upper band in bottom blot) was quantified through analysis with ImageJ and normalization against the Gin4–GFP signal. The level of phosphorylated-Gin4–GFP (Phos-Gin4) at each time point is shown under the blot. Scale bars: 5 µm.

Cdc14 is the effector of the MEN and remains sequestered in the nucleolus throughout most of the cell cycle until activation of the MEN triggers its release; Cdc14 also localizes to the bud neck in late anaphase (Clemente-Blanco et al., 2006; de Bettignies and Johnston, 2003). To determine if the NAD interacts with Cdc14, we first examined if they colocalized in *GIN4*-ON cells that coexpressed Cdc14–GFP with mCherry–CT3 or mCherry–Gin4^CT1Δ^. We found that Cdc14–GFP colocalized with mCherry–CT3 in the nucleus and with mCherry–Gin4^CT1Δ^ both in the nucleus and at the bud neck in a population of cells (Fig. 6A; Fig. S3C). Next, we determined whether the NAD physically associates with Cdc14. Cdc14–Myc was coexpressed with the various NAD-containing Gin4 constructs, GFP–Gin4, GFP–CT3, GFP–CT3.3 and GFP–Gin4^CT1Δ^. We performed coimmunoprecipitation in log-phase yeast cells by precipitating the GFP-tagged Gin4 proteins and probing Cdc14 with an antibody against Myc in a western blot. Cdc14 was detected in the pulldown products of all the Gin4 variants (Fig. 6B). CT3.3 interaction with Cdc14 was also detected in *GIN4*-OFF cells (Fig. S4B), although the amount precipitated was less than that from *GIN4*-ON cells, possibly because of the phenotypic consequences of Gin4 depletion. We also conducted coimmunoprecipitation analyses to investigate the interaction of Gin4 or Gin4^CT3Δ^ with Cdc14 and found that Cdc14 co-precipitated with both Gin4 and Gin4^CT3Δ^ in similar amounts in *GIN4*-ON and -OFF cells (Fig. S4C). The data suggest the presence of redundant mechanisms that are independent of the NAD and that mediate the association of Cdc14 with Gin4, possibly through components in the septin ring. Consistently, Cdc14 could still localize to the bud neck in *GIN4-*OFF *gin4^CT3Δ^* cells (Fig. S4A).

*Ca*Gin4 undergoes cell-cycle-dependent phosphorylation by Cdc28 upon entry into mitosis (Li et al., 2012). To show that Gin4 is a substrate of Cdc14, we purified GST–Cdc14 from *E. coli* and mixed it with immunoprecipitated Gin4–GFP in an *in vitro* phosphatase assay. Catalytically inactive Cdc14 (with the mutation C275S; Cdc14^C275S^) was included as a negative control (Bloom et al., 2011). Phosphorylated Gin4 was probed by western blotting with an antibody that specifically recognizes the CDK-phosphorylated serine residue (shown in bold) within CDK-target sites (**S**PXK/R; PScdk) (Greig et al., 2015; Li et al., 2012; Zheng et al., 2007). Exposure of Gin4–GFP to GST–Cdc14 markedly reduced the amount of phosphorylated Gin4, whereas the mock and GST-Cdc14^C275S^ conditions had no effect (Fig. 6C). Lambda phosphatase (λpp), as a positive control, significantly reduced the amount of phosphorylated Gin4. We examined the phosphorylation status of Gin4 in WT and *cdc14Δ/Δ* cells coexpressing GFP–Gin4 and Nop1–mCherry (as a nuclear marker) to determine whether Cdc14 dephosphorylates Gin4 *in vivo*. To obtain synchronous cultures, we grew cells to the stationary phase when >95% of the cells were in G1 and then released them into fresh medium for growth at 30°C. We harvested aliquots at intervals, and generated budding and nuclear division indices to compare cell cycle progression in the two strains (*n*>100). We observed only a slight delay in *cdc14Δ/Δ* cells at the end of the first cell cycle (180 min) (Fig. 6D), consistent with a previous report (Clemente-Blanco et al., 2006). We then immunoprecipitated Gin4–GFP and probed Gin4 for PScdk residues by western blotting. Both the WT and *cdc14Δ/Δ* strains contained untagged and GFP-tagged Gin4. Because Gin4 exists as dimers during mitosis (Mortensen et al., 2002), pulling down Gin4–GFP would also pull down untagged Gin4. Thus, blotting for GFP detected only Gin4–GFP, whereas blotting for PScdk detected phosphoserine residues in both Gin4 and Gin4–GFP, yielding two phosphorylated Gin4 bands (Fig. 6D). As previously reported (Li et al., 2012), levels of both Gin4 and its phosphorylation exhibited cell-cycle dependence in WT cells, peaking at 120–150 min; also, phosphorylated Gin4 was undetectable at 60 min (Fig. 6D). In comparison, phosphorylated Gin4 was readily detected at 60 min in *cdc14Δ/Δ* cells (Fig. 6D). Moreover, phosphorylated Gin4 levels in *cdc14Δ/Δ* cells were on average ∼1.7 times higher than in WT cells. Hence, we concluded that Cdc14 interacts with and dephosphorylates Gin4 *in vivo* and that the NAD likely mediates this interaction.

## DISCUSSION

The role of Nim1 protein kinases in cell cycle control is well-documented in the yeast models *S. pombe* and *S. cerevisiae*. However, to date, attention has been focused primarily on their kinase activity. Previously, we have discovered association of crucial functions with the large C-terminal non-kinase region of *Ca*Gin4 and proposed that Gin4 has both kinase-independent and -dependent activities, the former being essential for septin ring assembly and the latter having a role in mitosis (Li et al., 2012). In this study, we dissected and functionally characterized sub-fragments of the *Ca*Gin4 non-kinase region and revealed three distinct functional domains. Our findings shed new light on the molecular mechanisms by which Gin4 regulates septin assembly and mitosis.

### LBD – a domain that binds phospholipids and mediates association with the plasma membrane

Previous studies in both *S. cerevisiae* and *C. albicans* have reported Gin4 localization to specific cellular sites, such as the bud tip and the bud neck, in a cell-cycle-dependent manner. Here, we observed that the C-terminal 300 residues (CT1) of Gin4, when expressed alone, exhibited strong plasma membrane association. Although this is consistent with the plasma membrane localization of the KA1 domain found in the C-terminus of *Sc*Gin4, *Sc*Kcc1 and *Sc*Hsl1 (Moravcevic et al., 2010), we found that the region of *Ca*Gin4 that is homologous to the KA1 domain is not required for the localization of CT1 to the plasma membrane; instead, we mapped the plasma-membrane-targeting motif to the 100-amino-acid region (CT1.3) that is immediately N-terminal to the KA1 domain. The KA1 domain of *Sc*Kcc4, *Sc*Gin4 and *Sc*Hsl1 binds to phospholipids, especially phosphatidylserine (Moravcevic et al., 2010). We discovered that *Ca*CT1.3 binds to phosphoinositides. We were unable to test the interaction of *Ca*Gin4's KA1 domain with phospholipids because repeated efforts failed to obtain soluble GST–KA1 fusion protein. Although both CT1 and CT1.3 showed binding to phosphatidic acid and phosphatidylserine in the PIPstrip™ assays, the control GST exhibited strong binding to phosphatidic acid and phosphatidylserine as well. Nonetheless, we conclude that CT1.3 interacts with phosphoinositides and is crucial for the association of *Ca*Gin4 with the plasma membrane.

### SBD – a domain that directly associates with the septins

Septins and Gin4 colocalize throughout most of the cell cycle, and Gin4 is always abundantly immunopurified with the septin complex (Li et al., 2012; Mortensen et al., 2002), suggesting a direct Gin4–septin interaction. However, the Gin4 domain responsible for septin association has remained undefined. In yeasts, dozens of proteins associate with septins. Although Okuzaki et al. (1997) have identified a growth-inhibitory domain in *Sc*Gin4 that shows bud-neck localization, no general septin binding motifs have been reported. In this work, we identified that region 751–1050 of *Ca*Gin4 contains a potential SBD. This domain, when expressed in *GIN4*-ON cells, colocalizes with septins throughout the cell cycle and also coimmunoprecipitates with Cdc12. Mutant Gin4 that lacks the SBD localizes diffusely to the cytoplasm and fails to support normal septin ring assembly in *GIN4*-OFF cells. Also, the SBD can directly bind Cdc12 *in vitro*, indicative of a bona fide SBD. Despite its ability to bind to septins, septin ring assembly is not supported in *GIN4*-OFF cells that expressed the SBD alone, suggesting that a collaboration with other Gin4 domains is required. Indeed, cells expressing the CT2+1 fragment, encompassing only the SBD and LBD, assembled the septin ring and formed neck constrictions, albeit with cytokinesis defects. The formation of transient septin rings in the *GIN4*-OFF cells that expressed CT1 (Fig. 2A) suggested that CT1 might also be able to associate with the septins. Indeed, coimmunoprecipitation experiments confirmed this interaction, which, interestingly, occurs to a similar degree in *GIN4*-ON and *GIN4*-OFF cells (Fig. S1B). Because CT1 did not colocalize with the septin ring, the observation seems to suggest that CT1 might interact with septin molecules in the cytosol but not those in the ring. It is possible that CT1 has a chaperone-like function that mediates the interaction of Gin4 with the septins before septin ring assembly. Our data indicate that proper septin ring assembly requires the interaction of *Ca*Gin4 with both septins and phosphoinositides. Although septins have a lipid-binding motif near to the N-terminus and can self-assemble into filaments and higher-order structures upon contact with lipid membranes, the formation of the septin ring requires more than septin polymerization (Bridges and Gladfelter, 2015; Bridges et al., 2014). Here, we show that, at least in *C. albicans*, septin ring formation depends on the ability of Gin4 to interact with septins and phospholipids. We propose that the SBD-LBD region acts as a chaperone, creating the conditions required for septin ring assembly.

### NAD – a domain that localizes to the periphery of the nucleolus, associates with Cdc14 and has a possible role in mitotic exit

Gin4^CT1Δ^ localizes as a single or a pair of dots in the nucleus, and we identified the smallest motif showing the same localization at residues 551–650 (CT3.3). Although the dots exhibit SPB-like behavior, we found that they did not colocalize with the SPB marker Tem1 but instead localized to a site at the periphery of the nucleolus – the apparatus that sequesters the MEN effector Cdc14 (Segal, 2011; Visintin et al., 1998). Cells expressing Gin4 that lacked the NAD (*gin4^CT3^*^Δ^) showed frequent premature spindle elongation and nuclear division within the mother cell. Even though a previous study did not detect sequestration of *Ca*Cdc14 in the nucleolus (Clemente-Blanco et al., 2006), we observed colocalization of the NAD and Cdc14 in the periphery of the nucleolus in many cells. Also, coimmunoprecipitation experiments suggest that the NAD and Cdc14 physically associate. We hypothesize that the NAD-mediated interaction of Gin4 with Cdc14 could play a role in mitotic exit, although further investigations are necessary to unravel the exact mechanism. We were unable to detect full-length Gin4 in the nucleus. The nuclear localization of Gin4^CT1Δ^ suggests that full-length Gin4 might enter the nucleus only when its interaction with phospholipids is disrupted at certain stages of the cell cycle, which could be a transient event that affects only a small fraction of Gin4 molecules. Like the LBD of *Ca*Gin4, the association of the *Sc*KA1 domain with the plasma membrane is only revealed when truncated versions of the *Sc*Nim1 kinases are expressed (Moravcevic et al., 2010). Thus, further investigation is warranted to confirm whether full-length Gin4 localizes to the nucleus. However, we cannot rule out the possibility that the observed nucleolar association of the NAD could be an artifact that only occurs in some truncated versions of Gin4.

Cdc14 localizes to the bud neck during cytokinesis in *C. albicans* (Clemente-Blanco et al., 2006) and also preferentially dephosphorylates phosphorylated CDK sites (Queralt and Uhlmann, 2008; Stegmeier and Amon, 2004). *Ca*Gin4 has nine perfect CDK sites with several that are phosphorylated by Cdc28, making it a potential substrate of Cdc14. Consistently, Cdc14 coimmunoprecipitated with Gin4, and recombinant Cdc14 could dephosphorylate Gin4 *in vitro*. In *S. cerevisiae*, Gin4 also associates with Cdc14 (Bloom et al., 2011). Furthermore, in *cdc14Δ/Δ* mutants, *Ca*Gin4 phosphorylation was significantly higher than that in WT cells (Fig. 6E). Sanchez-Diaz et al. (2008) propose that Cdc14 dephosphorylation of CDK substrates in anaphase is central to the initiation of cytokinesis. Our data suggest that Gin4 could be an important Cdc14 target for this mitotic event.

**Fig. 7.**
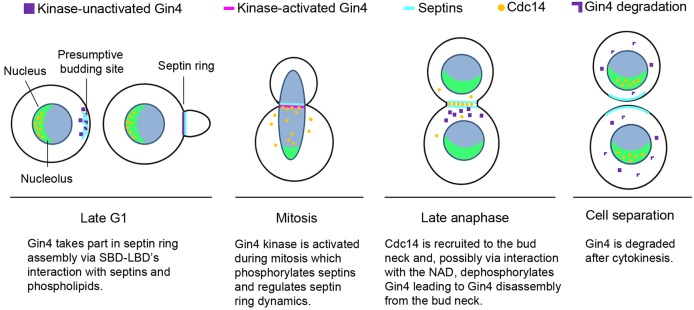
**A schematic description of the function of Gin4 during a cell cycle.**

In summary, our studies have uncovered multiple functional domains in the non-kinase region of Gin4. The findings provide new insights into how Gin4 regulates septin assembly and the associated cell cycle events. Fig. 7 presents a model describing the role of each Gin4 domain during the cell cycle. At the starting point, the LBD and SBD interact cooperatively with the septins and phospholipids at the presumptive bud site to initiate septin ring assembly at the bud neck. As the cell cycle progresses, Gin4 kinase activity is activated during mitosis, which stabilizes the septin collar (Li et al., 2012). In late anaphase, upon recruitment of Cdc14 to the bud neck, Cdc14 dephosphorylates Gin4, possibly through an interaction with the NAD, resulting in Gin4 disassembly from the septin ring. Then cytokinesis ensues, and Gin4 is degraded.

## MATERIALS AND METHODS

### Strains and culture conditions

All strains used in this study are listed in Table S1.

*C. albicans* strains were routinely grown at 30°C in yeast extract-peptone-dextrose medium (YPD) (2% yeast extract, 1% peptone and 2% glucose), or in glucose minimal medium (GMM) (2% glucose and 0.67% yeast nitrogen base without amino acids or with required amino acids). For *GIN4*-ON conditions, cells were cultured in maltose minimal medium (MalMM) (2% maltose and 0.67% yeast nitrogen base without amino acids or with required amino acids). For *GIN4*-OFF conditions, cells were grown overnight in GMM at 30°C and reinoculated at 1:10 dilution into fresh GMM for further growth. Hyphal induction was performed in liquid GMM or MalMM supplemented with 20% fetal bovine serum (FBS) and incubation at 37°C.

### Centrifugal elutriation

50 ml of an overnight yeast culture was reinoculated into 450 ml of fresh medium and grown to stationary phase. Cell synchronization was performed as described previously (Bensen et al., 2005).

### Plasmid construction for gene truncation, gene tagging and mutagenesis

The pClpGFP plasmid (Zheng et al., 2003) was modified to create the truncated *GIN4* mutant constructs. The 3′ UTR of *GAL4* was PCR-amplified with the addition of *Pst*I and *Mlu*I sites to the 5′ and 3′ ends, respectively, and cloned into the *Pst*I-*Mlu*I sites on pP*_MET3_*GFP to generate pP*_MET3_*GFPutr. The *GIN4* open reading frame was PCR-amplified from BWP17 genomic DNA using primers that added a *Nar*I site to the 5′ and a *Pst*I site to the 3′ ends, and was cloned into the *Cla*I*-Pst*I site on pP*_MET3_*GFPutr downstream of GFP to yield pP*_MET3_*GFP-GIN4. pP*_MET3_*GFP-GIN4 was linearized at a unique *Sal*I site within the *MET3* promoter for integration into the genome. pP*_MET3_*GFP–Gin4^CT2^^Δ^ and pP*_MET3_*GFP–Gin4^CT3^^Δ^ plasmids were generated by deleting the CT2 and CT3 regions, respectively, from pP*_MET3_*GFP–GIN4 using Quikchange™ site-directed mutagenesis kit (Agilent Technologies). The coding region for *gin4^CT1^*^Δ^, CT1, CT2 and CT3 of Gin4 were PCR-amplified using appropriate primers with *Nar*I and *Pst*I sites added at the 5′ and 3′ ends, respectively, and cloned into the *Cla*I*-Pst*I sites on pP*_MET3_*GFPutr.

The targeted gene or gene fragment was PCR-amplified and cloned into *Bam*HI*-Xho*I sites of pGEX-4T-1 (GE Life Sciences) to generate GST fusions. Mutation of the basic residues on CT1.3 and generation of the Cdc14^C274S^ mutant were performed using the Quikchange™ multi-site directed mutagenesis kit (Agilent Technologies) according to the manufacturer's instructions.

### Protein extractions, western blotting and coimmunoprecipitation

Protein work was performed as described previously (Li et al., 2012). Immunoprecipitation of GFP-tagged proteins were performed by using either the µMACS GFP Isolation Kit (Miltenyi Biotec) or the GFP-Trap (Chromotek). Protein extraction was performed using lysis buffer containing 150 mM NaCl, 1% Triton X-100, 50 mM Tris-HCl pH8.0, complete EDTA-free protease inhibitor cocktail tablets (Roche) and phosSTOP phosphatase inhibitor cocktail tablets (Roche). Protein concentration was estimated using the BCA Protein Assay kit (Thermo Scientific). Approximately 2 mg of protein was incubated with 50 µl of anti-GFP microbeads at 4°C for 2 h. The beads were then washed four times with low-salt wash buffer (150 mM NaCl, 0.1% Triton-X, 0.5% sodium deoxycholate, 0.1% SDS, 50 mM Tris-HCl, pH 7.2) and once with 20 mM Tris-HCl, pH 7.5, before elution with pre-warmed elution buffer (50 mM Tris-HCl pH 6.8, 50 mM DTT, 1% SDS, 1 mM EDTA, 10% glycerol and bromophenol blue). Wash with high-salt buffer (1 M NaCl) was performed three times, followed once by low-salt wash and once with 20 mM Tris-HCl, pH 7.5, before elution. The eluted proteins were separated on 10% SDS-PAGE gels, and western blotting was performed using appropriate antibodies. For immunoprecipitation of Gin4–GFP from WT (BWP17) and *cdc14Δ/Δ* cells, stationary phase cells were reinoculated at 1:20 dilution into pre-warmed fresh YPD medium. Aliquots of cells were then collected at timed intervals for immunoprecipitation as described above. Protein bands on western blots were quantified using ImageJ.

### Purification of GST-fusion protein

GST-fusion proteins (GST–CT1, GST–CT2, GST–Cdc14 and GST–Cdc14^C274S^) were expressed in BL21-CodonPlus (DE3)-RIL competent cells (Agilent Technologies). Plasmid-containing cells were cultured in 10 ml Luria-Bertani (LB)+ampicillin medium (100 µg/ml ampicillin) at 37°C overnight, reinoculated to 400 ml (1:100) LB+ampicillin medium and grown at 37°C to OD_600_=0.6–0.8. GST-fusion-protein expression was induced at 30°C overnight by adding 1 M IPTG to a final concentration of 0.5 mM. Cell pellets were collected and washed twice with 1× PBS. Protein was extracted by resuspending the pellet in five times the volume of lysis buffer (50 mM Tris-HCl pH 8.0, 150 mM NaCl, 1% Triton-X 100, 1% glycerol, 4 mM β-mercaptoethanol, 1 mM PMSF and Roche complete EDTA-free protease inhibitor cocktail), sonicated at 30% setting for 30 s and kept on ice for 2 min. The sonication was repeated six times, the cell debris was spun down at 15,000 rpm for 30 min at 4°C (Eppendorf 5424 microcentrifuge). The supernatant was collected and 500 ml of glutathione Sepharose 4B beads (GE Healthcare; prewashed with 1× PBS) were added. The mixture was incubated at 4°C on a roller overnight. Beads were collected using a column and washed three times with cold high-salt buffer (50 mM Tris-HCl, pH 8.0, and 300 mM NaCl) and once with cold low-salt buffer (50 mM Tris-HCl, pH 8.0, and 150 mM NaCl). GST-bound beads were incubated with 500 ml of elution buffer (50 mM Tris-HCl, pH 8.0, 150 mM NaCl and 20 mM reduced glutathione) for 45 min, and the supernatant was collected. Protein concentrations were determined using NanoDrop™ 1000.

### Protein lipid overlay assay

The interaction of GST–CT1 with phospholipids was investigated using the PIPStrip™ (Echelon Biosciences) according to manufacturer's protocol with slight modifications. The PIPstrips were blocked for 1 h in 1% skim dry milk in Tris-buffered saline with 0.1% v/v Tween-20 (TBS-T) at room temperature before incubation with purified GST–CT1 in 5% bovine serum albumin (BSA) in TBS-T at 4°C overnight. The strips were washed three times for 10 min with TBS-T and then probed with anti-GST monoclonal antibody (Santa Cruz) at a dilution of 1:1000, followed by anti-mouse-IgG peroxidase-conjugated antibody at a dilution of 1:5000 (Sigma-Aldrich). Both antibodies were prepared with 5% BSA in TBS-T. Enhanced chemiluminescence detection was performed with SuperSignal West Pico Chemiluminescent Substrate (Pierce-Thermo Scientific).

### *In vitro* phosphatase assay

*In vitro* phosphatase assays were performed in conditions as described previously (Bloom et al., 2011). GFP–Gin4 was immunopurified from log-phase cells (called JY56), and precipitates were washed four times with lysis buffer and then twice with 1× phosphatase buffer. λ-phosphatase (NEB) treatment was performed under the same conditions.

### Time-lapse and fluorescence microscopy

A Leica DMRXA2 microscope with 100× objective and a Hamamatsu digital camera interfaced with METAMORPH software (Universal Imaging) was used. Cell morphology and localization of fluorescent protein were visualized in living cells without fixing. Nuclei were stained using mounting medium with DAPI (Vectashield^®^). Time-lapse microscopy was performed on an inverted confocal laser LSM700 microscope (Carl Zeiss) with an attached temperature chamber and a photometrics coolsnap HQ2 digital camera interfaced with METAMORPH software (Universal Imaging). 1× GMM with 2% agarose or 1× MalMM with 2% agarose was spotted onto glass slides and cooled. Live cells were mounted onto the agar and covered with a cover slip and sealed. Image recoloring and sum projection of *z*-stacks was performed using Fiji (http://fiji.sc/Fiji).

### Confocal microscopy and FRAP analysis

Confocal microscopy and FRAP were performed using an Olympus Inverted Confocal microscope interfaced with FluoView Imaging System as described previously (Li et al., 2012). Analyses were performed with Fiji.
